# Identification of a novel necroptosis-associated miRNA signature for predicting the prognosis in head and neck squamous cell carcinoma

**DOI:** 10.1515/med-2022-0575

**Published:** 2022-10-25

**Authors:** Jiezhong Guan, Xinyu Liu, Kang Wang, Yiqun Jia, Bo Yang

**Affiliations:** Department of Prosthodontics, Hospital of Stomatology, Guanghua School of Stomatology, Sun Yat-Sen University, Guangdong Provincial Key Laboratory of Stomatology, Guangzhou, Guangdong, China; Stomatology Center, Shenzhen People’s Hospital, The Second Clinical Medical College of Jinan University, The First Affiliated Hospital of Southern University of Science and Technology, Shenzhen, Guangdong, China

**Keywords:** head and neck cancer, necroptosis, prognostic markers, bioinformatics, microRNA

## Abstract

Head and neck squamous cell carcinoma (HNSCC) is one of the most aggressive malignancies that have a poor prognosis. Necroptosis has been demonstrated in recent years to be a form of inflammatory cell death occurring in multicellular organism, which plays complex roles in cancer. However, the expression of necroptosis-related miRNAs and genes in HNSCC and their correlations with prognosis remain unclear. In this study, R software was used to screen differentially expressed miRNAs downloaded from The Cancer Genome Atlas. A prognostic model containing six necroptosis-related miRNAs (miR-141-3p, miR-148a-3p, miR-331-3p, miR-543, miR-425-5p, and miR-7-5p) was generated, whose risk score was validated as an independent prognostic factor for HNSCC. Target genes of the key miRNAs were obtained from TargetScan, miRDB, and miRTarBase, and 193 genes in the intersection of the three databases were defined as consensus genes. Kyoto Encyclopedia of Genes and Genomes and Gene Ontology analyses indicated that the composition of the tumor microenvironment as well as specific pathways may be closely related to necroptosis in HNSCC. Nine key genes were also obtained by the MCODE and cytoHubba plug-ins of Cytoscape: PIK3CD, NRAS, PTK2, IRS2, IRS1, PARP1, KLF4, SMAD2, and DNMT1. A prognostic model formed by the key gene was also established, which can efficiently predict the overall survival of HNSCC patients. In conclusion, necroptosis-related miRNAs and genes play important roles in tumor development and metastasis and can be used to predict the prognosis of HNSCC.

## 1 Introduction

Most head and neck cancers are derived from the mucosal epithelium in the oral cavity, pharynx, and larynx and are known collectively as head and neck squamous cell carcinoma (HNSCC) [1]. HNSCC is the sixth most common cancer worldwide, with 890,000 new cases and 450,000 deaths in 2018. The incidence of HNSCC continues to rise and is anticipated to increase by 30% (i.e., 1.08 million new cases annually) by 2030 [2]. Nowadays, the principal modalities of curative therapy for locally or locoregionally confined HNSCC are resection, radiation, and immunotherapy, but there is still treatment failure and unavoidable micrometastases [3]. Although immunotherapy does less harm to patients, it may cause immune-related adverse events, such as pneumonitis, colitis, or other organ injuries [4]. Considering the limitations of existing treatments, new therapeutic targets are needed to improve the clinical outcomes of HNSCC. Hence, reliable novel prognostic models are urgently required for the improvement of targeted therapies.

Necroptosis is a form of programmed necrosis or inflammatory cell death occurring in multicellular organism, which is regulated and controlled by many cellular and molecular mechanisms. Necroptosis cells are featured by morphological characteristics, such as incomplete cell membranes, crisis of intracellular energy metabolism, and release of inflammatory factors [5]. Moreover, necroptosis has a dual effect on cancer [6,7]. Recently, necroptosis has been proved as the necessary condition for cancer metastasis and immunosuppression, whose regulation has been considered to be a new target of cancer treatment [8].

With the deeper research of microRNAs (miRNAs, miRs) recently, the correlation between several functional miRNAs and the development of HNSCC has been further confirmed [9]. As an essential part of the microenvironment, miRNAs are intimately involved in the processes of epithelial to mesenchymal transition (EMT), secretion from fibroblasts, inflammation, survival, gene expression, and stemness, which are vital in the regulation of tumor cell viability and the initiation of tumor development, progression, and drug resistance [10]. Necroptosis can be triggered by a specific tumor microenvironment, while it can also regulate the tumor microenvironment, the process of which is mediated by several miRNAs including miR-7-5p (miR-7), miR-148a-3p, miR-141-3p, miR-331-3p, and so forth [6]. miR-7 is reported to induce necroptosis by targeting SLC25A37 and TIMM50 to work as a tumor-suppressive gene [11]. Long non-coding RNA (lncRNA)-107053293 regulated necroptosis by acting as a competing endogenous RNA (ceRNA) of miR-148a-3p [12]. However, the regulation effect of miR-148a-3p and miR-141-3p was contradictory in different cancers.

Given the existing findings, we know that necroptosis plays a vital role in the development of tumors and the antineoplastic process, which is regulated by several new epigenetically regulated miRNAs. Previous studies showed that there are necroptosis-related biomarkers for predicting the prognosis of various cancers [13,14,15,16,17], but the potential of necroptosis-related miRNAs in predicting the prognosis of patients with HNSCC remains unclear. In this study, we aim to reveal the underlying roles of necroptosis-related miRNAs in the onset and progression of HNSCC and construct a novel signature utilizing necroptosis-related miRNAs for predicting the prognosis.

In the present study, we performed a systematic study to determine the expression levels of necroptosis-related miRNAs in normal head-and-neck and HNSCC tissues, explore the prognostic value of these miRNAs, as well as predict the relevant key genes that affect the occurrence of HNSCC. The prognostic miRNAs and key genes, we obtained, may exert considerable impact on both the necroptosis-related process and the progression of HNSCC, which enables them to become potential therapeutic targets and to be considered for future investigations on HNSCC.

## 2 Methods and materials

### 2.1 Data collection and identification of differentially expressed necroptosis-related miRNAs

The RNA-seq of 546 (44 normal and 502 tumor) samples and miRNAs-seq of 569 (44 normal and 525 tumor) samples from the TCGA-HNSCC cohort with the clinical information were downloaded from the TCGA database (https://portal.gdc.cancer.gov/) on September 1, 2021. The GSE65858 dataset was directly downloaded from the GEO database (https://www.ncbi.nlm.nih.gov/geo/query/acc.cgi?acc=GSE65858) on September 1, 2021, which includes 270 tumor samples. In addition, its relevant clinical survival data were further retrieved from the GEO2R website (https://www.ncbi.nlm.nih.gov/geo/geo2r/? acc=GSE65858) of the GEO database on September 15, 2021. The clinical characteristics of the patients are shown in Table S1. We extracted 16 necroptosis-related miRNAs from the previous reviews, and they are presented in Table S2. The limma package was used to identify differentially expressed miRNAs (DEMs) with a *P* value < 0.05.

### 2.2 Development of the necroptosis-associated miRNA signature

Univariate Cox proportional hazards regression analysis was performed on DEMs with survival package of R software. DEMs were selected, and multivariate Cox stepwise regression analysis was performed on them with a survival package of R software. After multivariate Cox, prognostic miRNAs were obtained, and prognostic miRNAs with *P* < 0.05 were considered independent prognostic factors. The regulatory network of the prognostic miRNAs constructed with miRWalk and then STRING was visualized with Cytoscape software. After the prognostic miRNAs screened, a prognostic model was established, and we computed the risk scores for each patient. This study divided the patients into a high-risk group and a low-risk group based on the median value of the risk score. The Kaplan–Meier survival curves of both groups were estimated. Then, we plotted the receiver operating characteristic (ROC) curve to test whether the predictive ability of the model was reliable.

### 2.3 Independent prognostic analysis of the risk score

We extracted the clinical information of patients in the TCGA cohort and analyzed them with the risk score by Cox regression models.

### 2.4 Construction and validation of a predictive nomogram

A hybrid nomogram was built with the “rms” R package encompassing the developed miRNA signature and clinicopathological attributes to predict HNSCC patient overall survival (OS) (1-year, 2-year, and 3-year). Calibration curve, time–ROC curve, and decision curve analysis (DCA) were used to check the predictive power of nomogram.

### 2.5 Gene set enrichment analysis (GSEA)

GSEA (version v4.1.0, http://www.gsea-msigdb.org/gsea/downloads) was employed to scrutinize the Kyoto Encyclopedia of Genes and Genomes (KEGG) pathway analysis and Gene Ontology (GO) analyses between the high-risk group and the low-risk group based on the developed necroptosis-related miRNA signature.

### 2.6 Target gene prediction for key miRNAs

TargetScan, miRDB, and miRTarBase were used to predict the target genes of the key miRNAs. The overlapping genes from all of the databases were employed. To further improve the reliability of these results, we identified the overlapping target genes by using the Venn Diagram package of R software to obtain the consensus genes.

### 2.7 Functional enrichment analysis of consensus genes

To further clarify the roles that the consensus genes play in biological processes, we used the DAVID (https://david.ncifcrf.gov/) to perform KEGG pathway enrichment and GO functional annotation analyses.

### 2.8 Analysis of immune infiltration of tumor cells

The “GSVA” package was used to conduct the ssGSEA to calculate the scores of infiltrating immune cells and to evaluate the activity of immune-related pathways.

### 2.9 Construction of the protein-protein interaction (PPI) network and screening of the core network

We used STRING to construct a PPI network with the common genes whose confidence score was greater than or equal to 0.700, and the disconnected genes were hidden. The network was then input into Cytoscape software to screen key genes using the MCC algorithm of the Cytoscape cytoHubba plug-in. Meanwhile, the functional modules of the common genes were scored and screened out using the Cytoscape MCODE plug-in with the following criteria: degree cut-off = 2, haircut on, node score cut-off = 0.2, *k*-core = 2, and max. depth = 100.

### 2.10 Establishment and validation of a prognostic model formed by screened key genes

We directly established a prognostic gene model formed by those key genes and conducted the survival analysis of this new model and plotted the risk score curve, survival status map, and survival curve. The ROC curve was used as a criterion to show the predictive capability of these models. Independent prognostic analysis of the risk score was also conducted. For the validation studies, an HNSCC cohort from the GEO database (GSE140082) was employed. The expression of each necroptosis-related gene was also normalized by the “scale” function, and the risk score was then calculated by the same formula used for the TCGA cohort.

### 2.11 Statistical analysis

Data were analyzed using Bioconductor packages in R software, version 4.0.2. Normally and non-normally distributed variables were analyzed using the unpaired Student’s *t*-test and the Wilcoxon test, respectively. The Benjamini–Hochberg method was used to identify the differently expressed miRNAs, based on FDR. The sensitivity and specificity of the derive prognostic signatures for HNSCC in comparison to other clinicopathological were assessed using the ROC and DCA.

## 3 Results

### 3.1 Identification of DEMs between normal and tumor tissues

The expression levels of 16 necroptosis-related miRNAs were compared using The Cancer Genome Atlas (TCGA) data from 44 normal and 525 tumor tissues and we identified 6 DEMs (all *P* < 0.01). Among them, the miR-148a-3p was downregulated and the expressions of miR-141-3p, miR-331-3p, miR-543, miR-425-5p, and miR-7-5p were enriched in the tumor group, and the results were presented by heatmap (Figure S1).

### 3.2 Construction of prognostic miRNA-based signature

**Figure 1:**
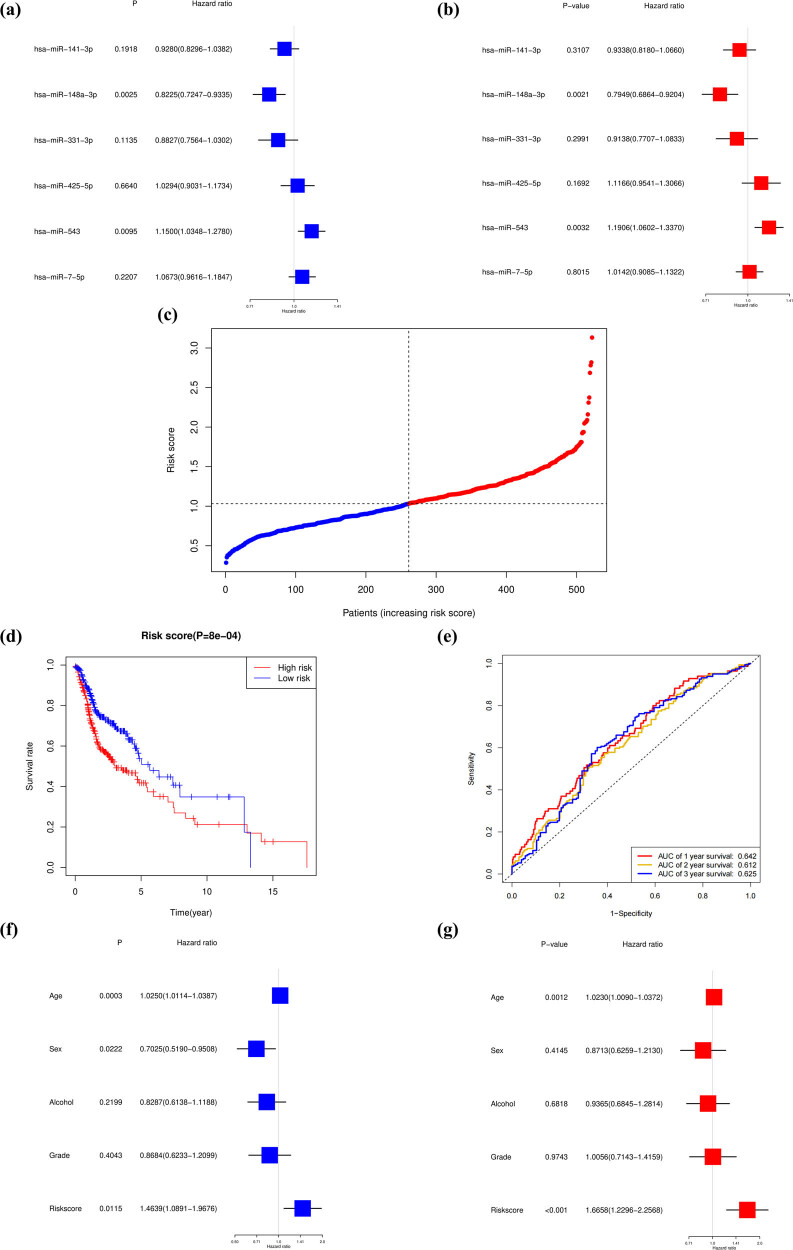
Construction of risk signature based on necroptosis-related miRNAs. (a) A univariate Cox regression analysis of OS for each necroptosis-related miRNA. (b) A multivariate Cox regression analysis of OS for each necroptosis-related miRNA. (c) Distribution of patients based on the risk score. (d) The Kaplan–Meier curve showed that the OS of the high-risk group was lower than that of the low-risk group. (e) The results of the ROC curve show that the model has an effective prediction ability. (f) Univariate analysis revealed that risk score was related to OS. (g) Multivariate analysis indicated that risk score was an independent prognostic factor for HNSCC.

Six miRNAs associated with the survival of HNSCC patients were identified and selected according to the univariate (Figure 1a) and multivariate (Figure 1b) Cox analysis together with the clinical significance. The previously published literature on the six miRNAs are summarized in Table 1 and the regulatory network of the prognostic miRNAs is constructed in Figure S2. Among them, the *P* values of hsa-miR-148a-3p were less than 0.05, indicating that it was the independent prognostic factor. The prognostic miRNA risk score was calculated according to the following formula:

\begin{array}{c}\text{Risk score}\hspace{0.25em}=\text{ }(-0.068\hspace{.25em}\text{⁎}\hspace{.25em}\text{exp}\text{. hsa}-\text{miR}-141-3\text{p})\\ \hspace{5.5em}+(-0.230\hspace{.25em}\text{⁎}\hspace{.25em}\text{exp}\text{.hsa}-\text{miR}-148a-3\text{p})\\ \hspace{5.5em}+(-0.090\hspace{.25em}\text{⁎}\hspace{.25em}\text{exp}\text{.hsa}-\text{miR}-331-3\text{p})\\ \hspace{5.5em}+(0.110\hspace{.25em}\text{⁎}\hspace{.25em}\text{exp}.\text{hsa}-\text{miR}-425-5\text{p})\\ \hspace{5.5em}+(0.174\hspace{.25em}\text{⁎}\hspace{.25em}\text{exp}.\text{hsa}-\text{miR}-543)\\ \hspace{5.5em}+(0.014\hspace{.25em}\text{⁎}\hspace{.25em}\text{exp}.\text{ hsa}-\text{miR}-7-5\text{p}).\end{array}]

**Table 1:** Literature associated with the six necroptosis-related miRNAs

miRNAs	Literature
miR-148a-3p	Wang W, et al. Ammonia regulates chicken tracheal cell necroptosis via the lncRNA-107053293/MiR-148a-3p/FAF1 axis. J Hazardous Mat. 2020;386:121626. doi: 10.1016/j.jhazmat.2019.121626
miR-141-3p	Li M, et al. miR-141-3p promotes proliferation and metastasis of nasopharyngeal carcinoma by targeting NME1. AdvMed Sci. 2020;65(2):252–8. doi: 10.1016/j.advms.2020.03.005
miR-425-5p	Gu C, et al. miR-425-5p improves inflammation and septic liver damage through negatively regulating the RIP1-mediated necroptosis. Inflamm Res Off J Europ Histamine Res Soc. 2020;69(3):299–308. doi: 10.1007/s00011-020-01321-5
miR-7-5p	Xiao H. MiR-7-5p suppresses tumor metastasis of non-small cell lung cancer by targeting NOVA2. Cellular Mol Biol Lett. 20 Nov. 2019;24(60). doi: 10.1186/s11658-019-0188-3
miR-543	Visalli M et al. miRNA expression profiling regulates necroptotic cell death in hepatocellular carcinoma. IntJ Oncol. 2018;53(2):771–80. doi: 10.3892/ijo.2018.4410
miR-331-3p	Yu C-H, et al. A combination of mRNA expression profile and miRNA expression profile identifies detection biomarkers in different tumor stages of laryngeal squamous cell carcinoma. European Rev Med Pharmacol Sci. 2018;22(21):7296–304. doi: 10.26355/eurrev_201811_16266

Then, the samples were divided into a high-risk group and a low-risk group based on the medium risk score. Figure 1c presents the detailed information on the risk score. Kaplan–Meier survival analysis showed that compared with the high-risk group, the low-risk group survival rate was higher (*P* = 8 × 10^−4^) (Figure 1d). The area under the ROC curve (AUC) was 0.642, 0.612, and 0.625 for 1-year, 2-year, and 3-year survival (Figure 1e), indicating that the model had moderate predictive efficacy. We used univariate and multivariate Cox regression analyses to evaluate whether the risk score derived from the gene signature model could serve as an independent prognostic factor, which implied that the risk score was an independent factor predicting poor survival (*P* = 0.0115, HR = 1.4639, 95% CI = 1.0891–1.9676) in univariate analysis (Figure 1f). The multivariate analysis also indicated that, after adjusting for other confounding factors, the risk score was an independent prognostic factor (*P* = 0.0078, HR = 1.5118, 95% CI 1.1150–2.0498) for patients with HNSCC (Figure 1g).

### 3.3 Construction and detection of the predictive nomogram

**Figure 2:**
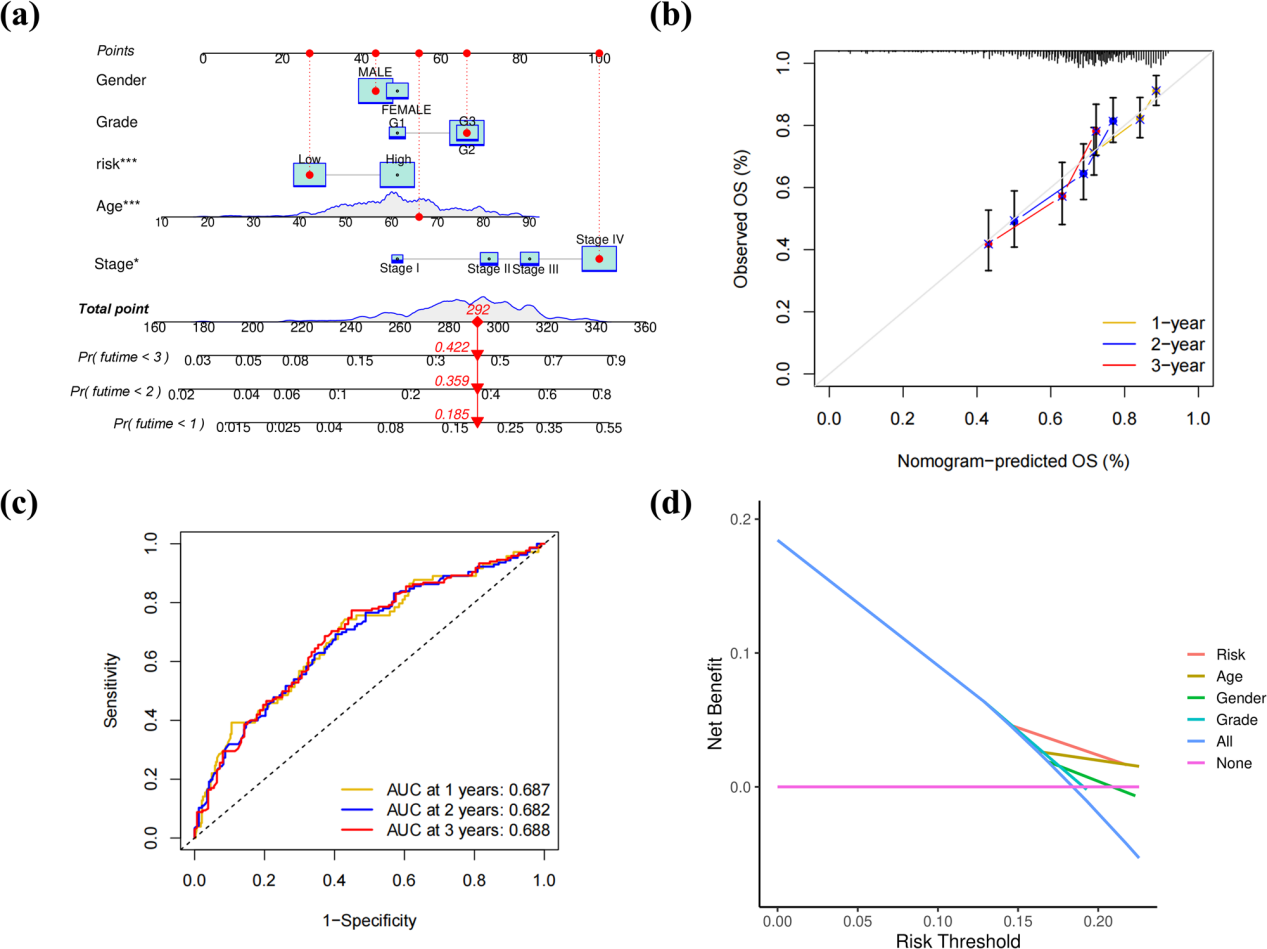
Construction and detection of the predictive nomogram. (a) Nomogram plot was built based on the prognostic factors in the whole cohort. (b) Calibration curve of the nomogram. (c) AUC of time-dependent ROC curves verified the prognostic accuracy of the nomogram. (d) The DCA curve of the prognostic factors indicated that risk score was the most critical factor for HNSCC.

To establish an effective methodology for predicting the survival probability of patients with HNSCC, we constructed a nomogram to predict the probability of 1-year, 2-year, and 3-year OS. The predictors of the nomogram incorporated clinical features and the risk score (Figure 2a). Calibration curve and time–ROC curve indicated that the stability and accuracy of this hybrid nomogram encompassing our miRNA signature with clinical features can eloquently function in clinically managing HNSCC patients (Figure 2b and c). DCA demonstrated that the risk model showed the best net benefit for predicting OS (Figure 2d).

### 3.4 GSEA based on the developed signature

**Figure 3:**
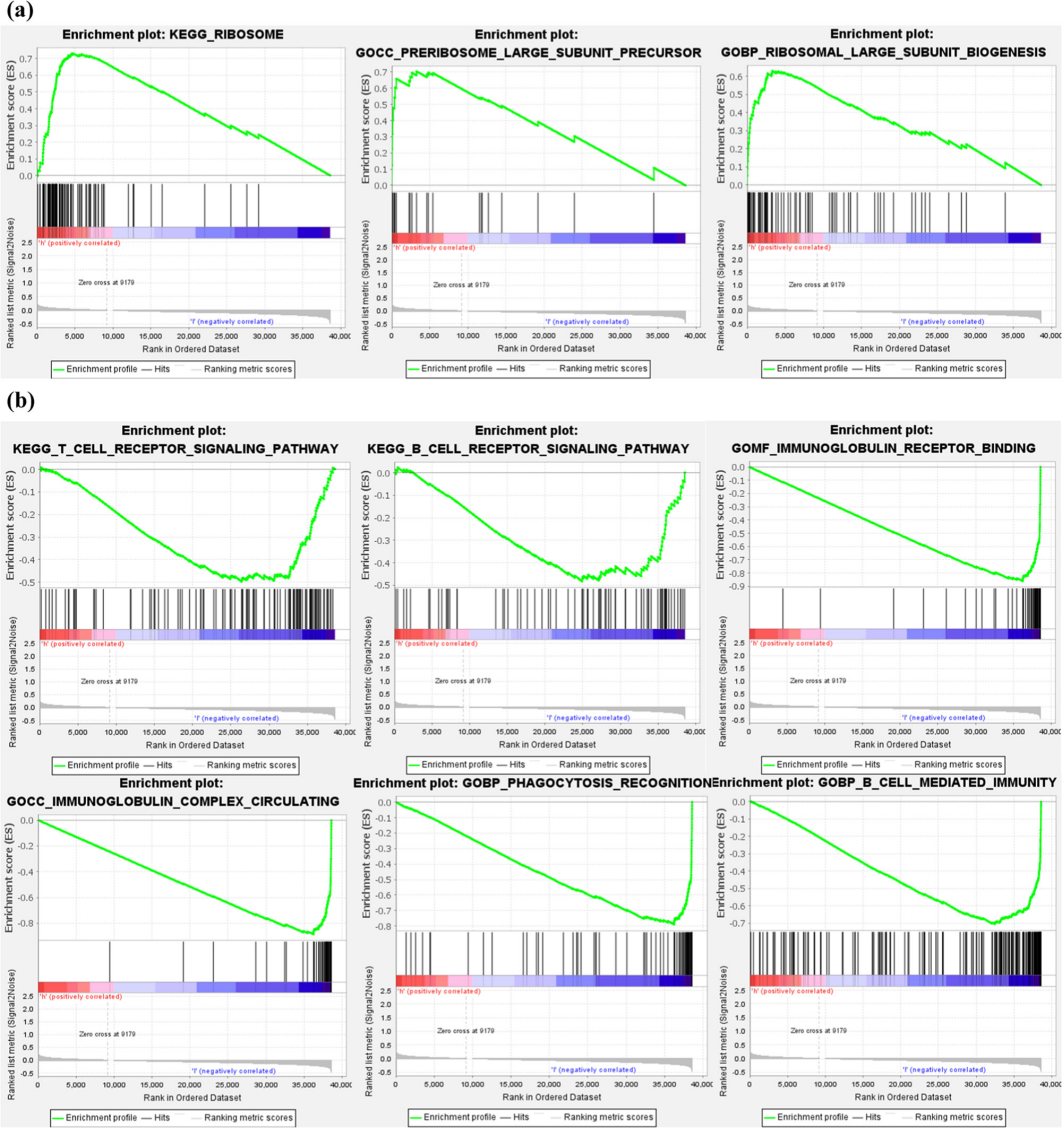
GSEA based on the developed signature. (a) GSEA results illustrate significant enrichment of ribosome-related pathways in the high-risk HNSCC patients. (b) GSEA results illustrate significant enrichment of immunoregulatory pathways against tumor in the low-risk HNSCC patients.

KEGG and GO analyses employing GSEA were carried out based on the necroptosis-related miRNA signature. In the high-risk HNSCC patient group, upregulation of ribosome-related pathways incorporating KEGG: ribosome, GO: preribosome large subunit precursor, and GO: ribosome large subunit biogenesis was observed (Figure 3a). An eye-opener was the obvious upregulation of several immune pathways in the low-risk HNSCC patient group. These included KEGG: T cell receptor signaling pathway, KEGG: B cell receptor signaling pathway, GO: immunoglobulin receptor binding, GO: immunoglobulin complex circulating, GO: phagocytosis recognition, and GO: B cell-mediated immunity (Figure 3b).

### 3.5 Comparison of the immune activity between subgroups

**Figure 4:**
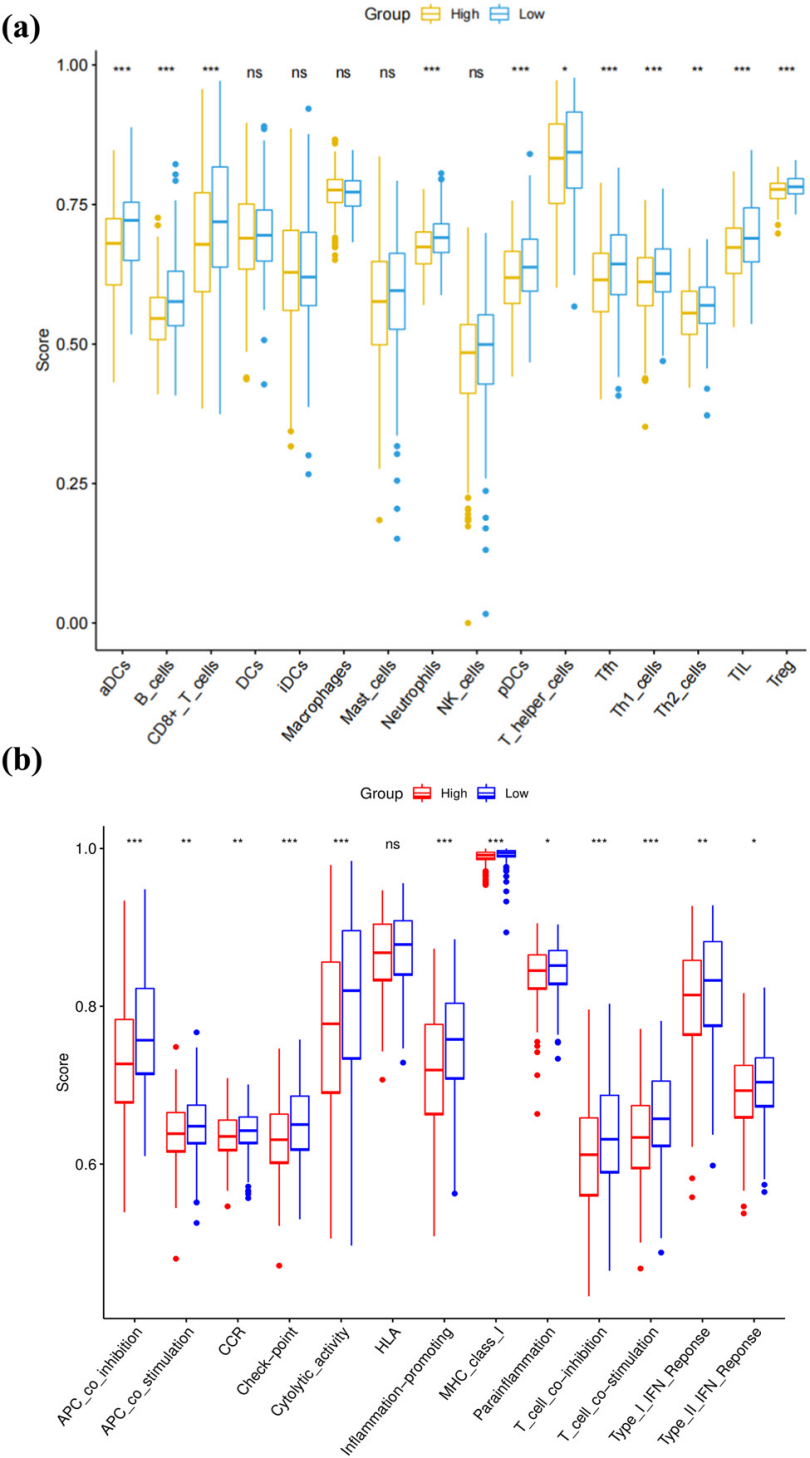
Analysis of immune infiltration of tumor cells. (a) Comparison of the ssGSEA scores for immune cells (high-risk group: red box and low-risk group: green box). (b) Comparison of the ssGSEA scores for immune pathways (high-risk group: red box and low-risk group: blue box).

Based on single-sample gene set enrichment analysis (ssGSEA) for immune infiltration analysis, we calculated the enrichment fractions of 16 kinds of immune cells and 13 immune-related pathways in the low-risk and high-risk groups to further compare the immune activity between subgroups. The results showed that the low-risk groups generally had higher levels of infiltration of immune cells than the high-risk groups, especially of dendritic cells (aDCs), B cells, CD8+ T cells, plasmacytoid dendritic cells (pDCs), T helper (Th) cells (Tfh, Th1, and Th2 cells), tumor-infiltrating lymphocytes (TILs), and regulatory T (Treg) cells (Figure 4a). Thirteen immune pathways showed higher activity in the low-risk group than in the high-risk group (Figure 4b), indicating that the activation of immune pathways in the high-risk group was poor.

### 3.6 OS analysis of six miRNAs and the target gene prediction of the key prognostic miRNAs

**Figure 5:**
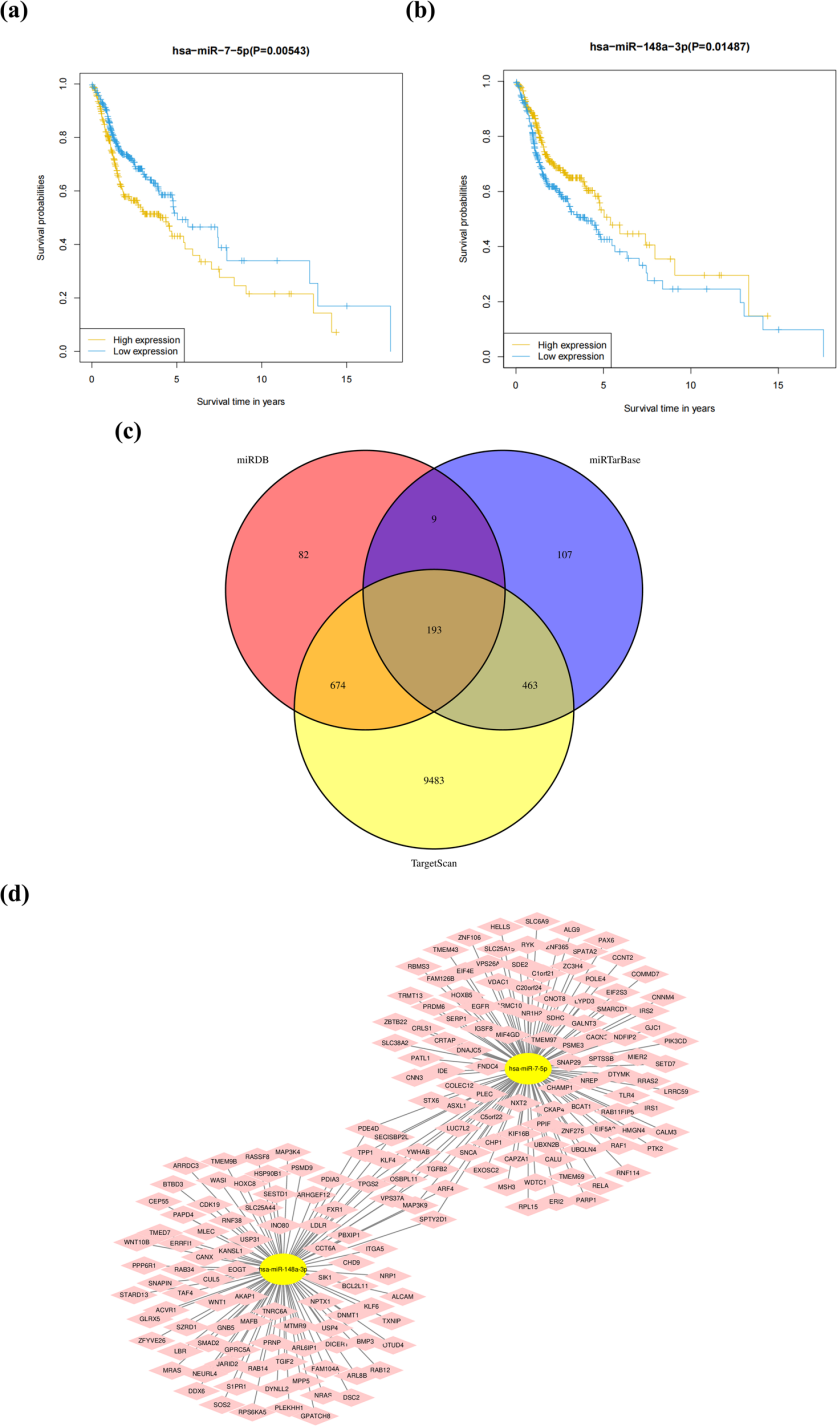
Survival analysis and the target genes prediction of the key prognostic miRNAs. (a) Kaplan–Meier curves for the OS of patients in the high and low expression groups of miR-7-5p. (b) Kaplan–Meier curves for the OS of patients in the high and low expression groups of miR-148a-3p. (c) The Venn figure shows the number of consensus genes based on online databases TargetScan, miRDB, and miRTarBase. (d) The miRNA-gene pathway and annotation networks represent the relationships among miRNAs and consensus genes.

We analyzed the expression of five miRNAs on patient survival by using the Kaplan–Meier curve. It was found that the expression of hsa-miR-7-5p (*P* = 0.00543) and hsa-miR-148a-3p (*P* = 0.01487) significantly affected the OS outcomes. The survival probability of the patients with high expression of hsa-miR-7-5p (Figure 5a) and low expression of has-miR-148a-3p (Figure 5b) was higher than that with low expression. The three online website databases were used to predict the target genes of the two key miRNAs, and a total of 12,602 target genes were obtained (10,969 and 1,633 target genes of hsa-miR-7-5p and hsa-miR-148a-3p, respectively). After excluding duplicate target genes that were jointly regulated by multiple miRNAs and taking the intersection of the three databases, 193 consensus genes were identified (Figure 5c). Cytoscape was applied for the visualization of the miRNA-gene network (Figure 5d).

### 3.7 Functional enrichment analysis of consensus genes

**Figure 6:**
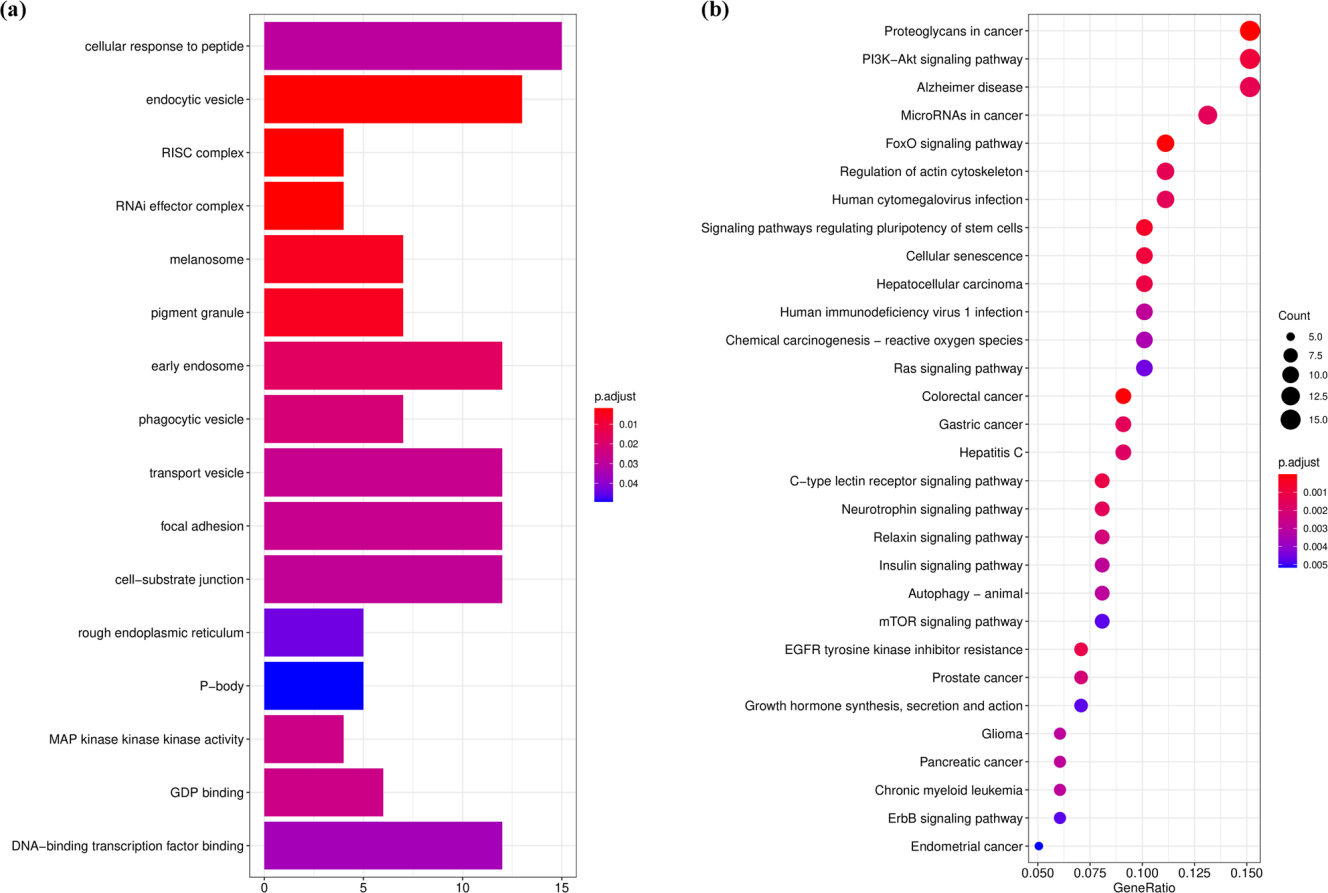
Functional enrichment analysis of consensus genes. (a) Bar plot graph for GO pathways (the longer bar means the more genes enriched, and the increasing depth of red means the differences were more obvious). (b) Bubble graph for KEGG enrichment (the bigger bubble means the more genes enriched, and the increasing depth of red means the differences were more obvious; *q*-value: the adjusted *P*-value).

Based on the GO enrichment and KEGG analysis, 16 GO terms were noticeably enriched with these 193 consensus genes that included cellular response to peptide, endocytic vesicle, and RISC complex, among others (Figure 6c). Forty-six KEGG pathways were noticeably enriched, including proteoglycans in cancer, PI3K-Akt signaling pathway, FoxO signaling pathway, and regulation of actin cytoskeleton, among others (Figure 6d).

### 3.8 PPI network construction and exploration of the key genes

**Figure 7:**
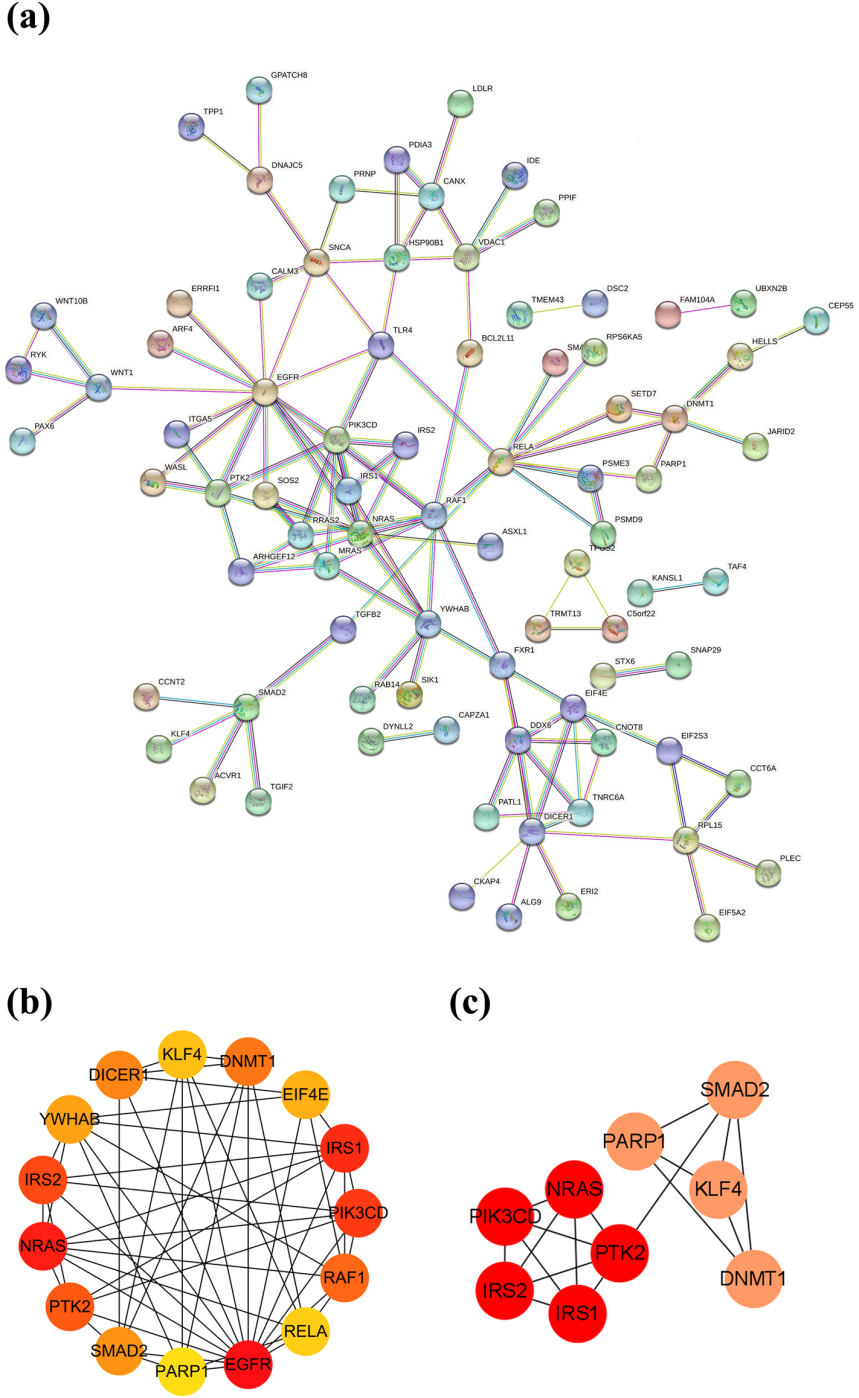
PPI network construction and exploration of the key genes. (a) PPI network of 193 consensus genes. (b) Results of the cytoHubba topological analysis. (c) MCODE network module diagram showed the key genes and their connections.

To study their PPIs, we entered all the 193 consensus genes into the STRING database to construct the PPI network. Next, for further analysis, we imported the genes with confidence scores above 0.75 into Cytoscape (Figure 7a). Using the MCC algorithm of the cytoHubba plug-in, the top 15 genes were identified (Figure 7b). The MCODE plug-in revealed two important functional modules in the interaction network that included nine key genes: PIK3CD, NRAS, PTK2, IRS2, IRS1 (MCODE score = 5.000), PARP1, KLF4, SMAD2, and DNMT1 (MCODE score = 4.000) (Figure 7c).

### 3.9 Establishment and validation of a prognostic model based on the key genes

We further placed the nine key genes into one prognostic model based on a total of 502 HNSCC patients in the training set of the TCGA database, and its prognostic risk score was calculated according to the following formula:

\begin{array}{c}\text{Risk score}\hspace{0.25em}=\hspace{0.25em}(-0.361\hspace{.25em}\ast \hspace{.25em}\text{exp}.\text{DNMT}1)+(0.052\hspace{.25em}\ast \hspace{.25em}\text{exp}.\text{IRS}1)\\ \hspace{5.5em}+(0.150\hspace{.25em}\ast \hspace{.25em}\text{exp}.\text{IRS}2)+(-0.086\hspace{.25em}\ast \hspace{.25em}\text{exp}.\text{KLF}4)\\ \hspace{5.5em}+(0.350\hspace{.25em}\ast \hspace{.25em}\text{exp}.\text{NRAS})+(0.186\hspace{.25em}\ast \hspace{.25em}\text{exp}.\text{PARP}1)\\ \hspace{5.5em}+(0-0.208\hspace{.25em}\ast \hspace{.25em}\text{exp}.\text{PIK}3\text{CD})\\ \hspace{5.5em}+(-0.010\hspace{.25em}\ast \hspace{.25em}\text{exp}.\text{PTK}2)+(-0.202\hspace{.25em}\ast \hspace{.25em}\text{exp}.\text{SMAD}2).\end{array}]

**Figure 8:**
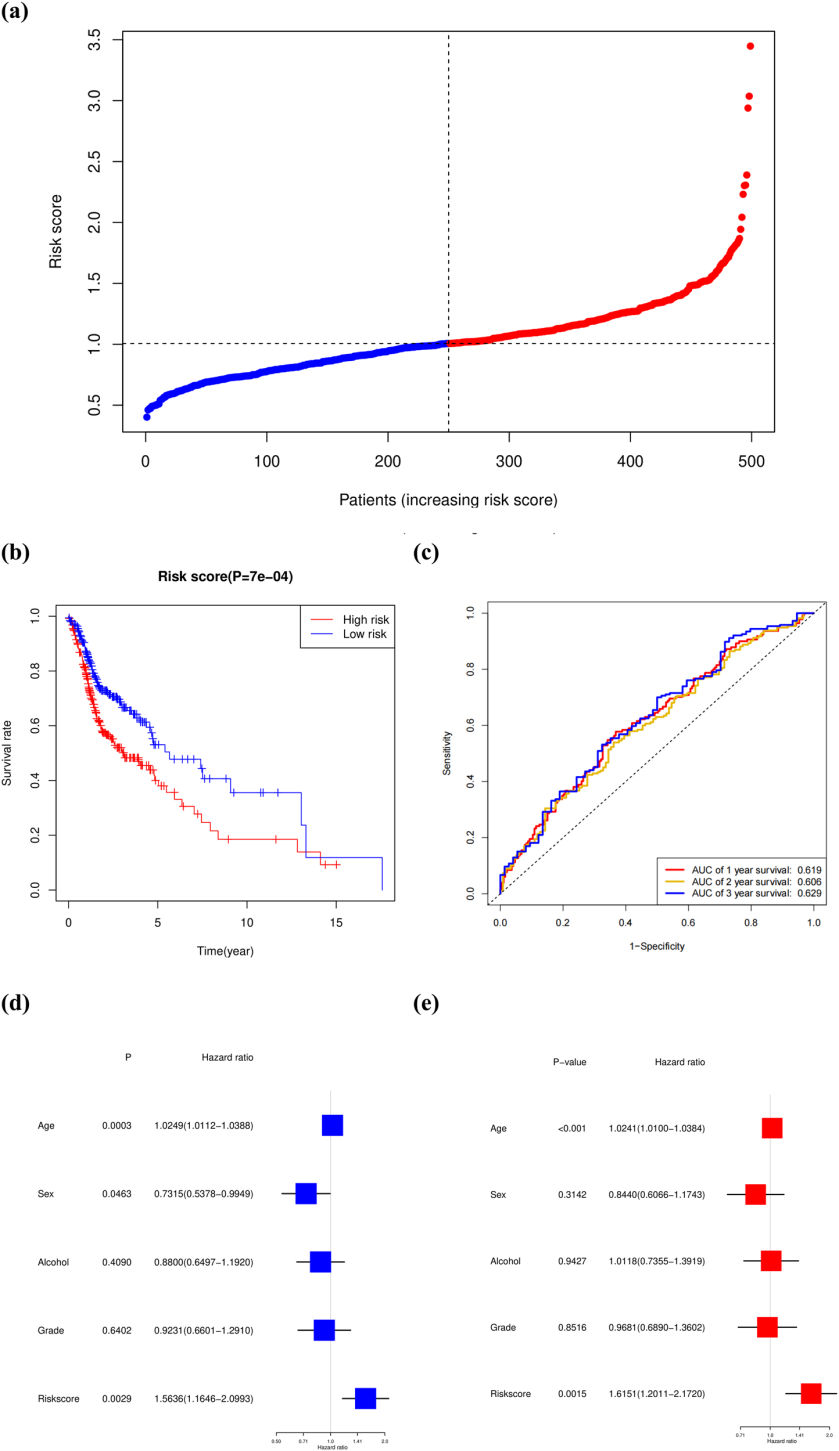
Establishment and validation of a prognostic model based on the key genes. (a) Distribution of patients based on the risk score. (b) Kaplan–Meier curves for the survival rate of patients in the high-risk and low-risk groups based on TCGA database. (c) Time-dependent ROC curves for OS. (d) Univariate Cox regression analysis of OS for five risk-related factors. (e) Multivariate Cox regression analysis of OS for five risk-related factors.

Then, the samples were divided into a high-risk group and a low-risk group based on the medium risk score. The risk score curve is plotted in Figure 8a. The high-risk score was correlated with a poor prognosis. Survival analysis showed that the mortality rate increased as the risk score increased. Compared with the high-risk group, the low-risk group survival rate was notably higher (*P* = 7 × 10^−4^) (Figure 8b). The ROC curve (AUC) was 0.619 for 1-year, 0.606 for 2-year, and 0.629 for 3-year survival (Figure 8c). Univariate and multivariable Cox regression analyses of the risk score derived from the gene signature model could serve as an independent prognostic factor predicting poor survival in both the training and testing sets (Figure 8d and e).

The credible predictive capability of the prognostic model was validated in an independent testing set of the GEO cohort including 270 HNSCC patients, indicating that the nine key genes were strongly associated with the prognosis of HNSCC (Figure S3).

## 4 Discussions

In the present study, we first studied the expression of 16 currently known necroptosis-related miRNAs in HNSCC and normal tissues. Then, we constructed a six miRNAs risk signature based on univariate and multivariable Cox regression analyses as well as the clinical significance to further assess the prognostic value of these necroptosis-related regulators. After two key miRNAs were obtained from the OS analysis, 12,602 target genes of the key miRNAs were obtained from TargetScan, miRDB, and miRTarBase prediction, and 193 genes in the intersection of the three databases were defined as consensus genes.

Necroptosis is a type of programmed cell death that depends on a unique molecular pathway, which is mediated by several intracellular signaling molecules such as receptor-interacting protein 1 (RIP1), RIP3, and mixed lineage kinase domain-like (MLKL). [18] Recent studies also provided novel insights into the function of miRNAs for the development of HNSCC [19]. For instance, tu006Dor suppression in HNSCC was suggested to be connected with miR-375-3p and miR-1229-3p [20,21,22]. Overexpression of miR-99a-5p inhibited the survival, proliferation, migration, and invasion of oral cancer cells [23]. Besides, miR-9 may serve as a valuable biomarker to identify the HNSCC patients who might benefit from RT + CTX (radiotherapy plus the anti-EGFR monoclonal antibody Cetuximab) therapy [24]. However, how necroptosis-associated genes function in HNSCC and whether they are related to patient survival remained unclear.

miR-141-3p is closely associated with necroptosis and plays a dual role in cancer. miR-141-3p can inhibit the upregulation of necroptosis-related molecules including IL-6 and TNF-α through direct interaction with RIPK1 [25]. Our results showed that upregulation of miR-141-3p may inhibit the release of necroptotic factors in HNSCC tumor microenvironment or promote metastasis of cancer cells. miR-331-3p and miR-425-5p play an important role in cell necroptosis in various cancers. The overexpression of miR-331-3p inhibits nasopharyngeal carcinoma cell proliferation through the elF4B-PI3K-Akt signaling pathway [26]. It has been found that miR-425-5p can directly target the 3′-UTR of RIP1 mRNA to reduce the expression of RIP1 and thus negatively regulate RIP1-mediated necroptosis [27]. Our results indicate that the high expression of miR-425-5p may impose positive effects on tumor progression via regulating cell necroptosis.

Among the six miRNAs, overexpression of miR-148a-3p and downregulation of miR-7-5p expression that were confirmed to be closely related to HSNCC in our prognostic model may affect the proliferation, migration, and invasion of HSNCC cells by regulating cell necroptosis.

miR-7-5p is characterized as a necroptosis promoter in cancer. In rhabdomyosarcoma, miR-7 is reported to downregulate TIMM50 and SLC25A37 and thus promote mitochondrial dysfunction, resulting in cell necroptosis [11]. TIMM50 and SLC25A37 are involved in inner mitochondrial membrane translocation, and their downregulation will lead to membrane damage and decreased oxygen consumption [11,28]. Interestingly, miR-7-5p seems to play a dual role in cancer progression. For instance, upregulation of miR-7-5p is responsible for the prohibition of oncogenic signaling in osteosarcoma [29] and melanoma [30]. However, its downregulation is confirmed to significantly induce apoptosis of esophageal cancer cells, thereby inhibiting their proliferation and metastasis [31]. Similarly, our result suggests that miR-7-5p may serve as a tumor promoter in HNSCC via regulating tumor necroptosis.

Contrary to miR-7-5p, miR-148-3p is identified to inhibit necroptosis. It has been demonstrated that lncRNA-107053293 functioned as a ceRNA of miR-148a-3p that regulated cell necroptosis by targeting FAF1, which mediates the downstream expression of RIPK1 and RIPK3, causing increased cell necroptosis [12]. As for its role in tumor development, miR-148-3p is reported as a tumor suppressor, as the upregulation of miR-148a-3p can target c-Jun mRNA to inhibit c-Jun protein expression and promote apoptosis of hepatocellular carcinoma cells infected with HCV [32]. Overexpression of miR-148a-3p can inhibit the occurrence of gastric cancer by inhibiting the hyaluronidase 1 gene and promoting cell apoptosis [33]. Our result is consistent with the abovementioned studies, suggesting that miR-148a-3p may regulate necroptosis through RIPK1/3-mediated pathway in HNSCC cells.

GSEA showed, in the high-risk HNSCC patient group, that ribosome-related pathways were upregulated, implying that it was closely connected to the poor prognosis of HNSCC, which was sustained by the previous experiments [34]. In the low-risk group, immune pathways such as T cell receptor signaling pathway and B cell receptor signaling pathway were upregulated, which were proven to play significant roles in the suppression of HNSCC [35,36]. Moreover, cell phagocytosis and immunoglobulin are widely applied in tumor immunotherapy [37,38], and related pathways were enriched in the low-risk group. The immune infiltration analysis indicates that the activation of immune cells and immune pathways in the low-risk group was higher than the high-risk group. Among them, immune cells such as B cells, CD8+ T cells, Th1 cells, TILs, and immune pathways such as APC-co-stimulation, cytolytic activity, MHC class I, and T-cell-co-stimulation play an inhibitory role in the occurrence and development of HNSCC [36,39,40,41,42,43,44].

By analyzing accessible online databases, we analyzed the potential of target genes and functions of the above two miRNAs. In the KEGG pathway analysis, a pathway related to proteoglycans in cancer and the PI3K-Akt signaling pathway were enriched. It was reported that PI3K-mediated tumor necrosis factor induced necroptosis through initiating RIP1–RIP3–MLKL signaling pathway activation, indicating that the PI3K-Akt signaling pathway may be essential for necroptosis initiation [45]. Regarding the result of GO analysis, endocytic vesicle was the functional annotation with most significance. Previous study implied that endocytic vesicle was associated with MLKL, the protein that mediates necroptosis, which controlled the transport of endocytosed proteins, thereby enhancing degradation of receptors and ligands, modulating their induced signaling and facilitating the generation of extracellular vesicles [46].

The functional analysis indicated that necroptosis can be regulated by the composition of the tumor microenvironment as well as specific pathways, and proteoglycans in cancer, PI3K-Akt signaling pathway, and endocytic vesicle will become the key to study the mechanism of necroptosis in HNSCC.

Finally, the consensus genes were analyzed with STRING and Cytoscape plug-ins, and nine key genes (PIK3CD, NRAS, PTK2, IRS2, IRS1, PARP1, KLF4, SMAD2, and DNMT1) were further acquired. We also constructed a prognostic model formed by these nine key genes, which was then validated to perform well in an external dataset.

Among the key genes, PIK3CD, NRAS, PTK2, IRS1, and IRS2 were enriched as a function module. The overexpression of PIK3CD, NRAS, PTK2, and IRS2 has been experimentally confirmed in HNSCC [47], while that of IRS1 is contradictory.

NRAS was suggested to be related to necroptosis, whose malfunction is strongly related to tumorigenesis and thus regarded as an important therapeutic target [48]. Therefore, miR-148a-3p in extracellular vesicles may regulate the metastatic potential of osteosarcoma cell lines by potentially inhibiting a network of genes that includes NRAS [49]. PIK3CD is the encoding gene of the catalytic subunit p110δ that forms PI3Kδ (phosphatidylinositol 3-kinase) complex together with a regulatory subunit [50]. We hypothesize that PIK3CD may be involved in the regulation of cell motility and invasion in HNSCC. Protein tyrosine kinase 2 (PTK2), encoding focal adhesion kinase (FAK), and FAK activation can increase cell migration via PI3K-mediated pathway. Its downregulation can result from overexpressing miR-138, which reduced cell motility and invasion colonies in HNSCC [51]. Insulin receptor substrates (IRS)-1 and IRS-2 are characterized as typical cytosolic adaptor proteins that involve insulin receptor (IR) and insulin-like growth factor I receptor (IGF-IR) signaling [52]. It seems that miR-7-5p can inhibit oncogenic functions of IRS-2. Our result supports the opposite, which may due to different types of cancer and requires further exploration.

The other four of the key genes (PARP1, KLF4, SMAD2, and DNMT1) were also functionally enriched. The four genes have been found to influence tumor progression in HNSCC via necroptosis regulation; Poly (ADP-ribose) polymerase-1 (PARP1), as a chromatin-associated enzyme in DNA repair; and a downstream effector of RIPK1/RIPK3 pathway in necroptosis [53]. DNA (-cytosine-5)-methyltransferase 1 (DNMT1) has been reported for silencing TNFα-RAPK1 and RIPK3-mediated necroptosis in cancer cell lines through hypermethylation [54,55]. It is confirmed that downregulation of miR-7-5p could significantly induce apoptosis of esophageal cancer cells by suppression of binding to Krüppel-like factor 4 (KLF4) [31]. In this study, we speculate a connection between inhibition of miR-148-3p and activation of SMAD2 and DNMT1, which is consistent with the previous studies [56,57]. Taken together, all four genes are strongly connected to TNFα/RIPK1-mediated necroptosis, indicating that these genes may also play a crucial role in regulating necroptosis in HNSCC. For the three genes (SMAD2, DNMT1, and KLF4) mentioned above, their role in tumor progression and necroptosis is opposite, suggesting necroptosis may act as the “insurance” that prevent tumor metastasis and invasion.

Interestingly, summing up the previous research studies, we found that the nine genes seemed to be closely associated with two pathways – RIP1-RIPS-MLKL (mixed-lineage kinase domain-like protein) pathway and RIPK1-RIPK3-MLKL pathway. Both the pathways affect MLKL trafficking and accumulation at the plasma membrane, which controls the kinetics and threshold for necroptosis [58].

Of importance, proteomic analysis identified PTK2 overexpression that can be used to evaluate cell tolerance to radiotherapy in locally advanced HNSCC. Therefore, combinations of PTK2/FAK inhibition with radiotherapy merit further evaluation as a therapeutic strategy for improving local in HPV-negative HNSCC [59]. Besides, PTK2 may potentially play a crucial role in determining the sensitivity of HNSCC to erlotinib clinically [60]. Furthermore, PARP1 inhibition can enhance the radiation sensitivity by disabling the DNA replication fork elongation response [61], which leads to deficiency of homologous recombination. There is also a phase I study suggesting that a PARPi-FL swish and spit solution is a rapid and noninvasive diagnostic tool that preferentially localizes fluorescence, contrasting with OSCC [62].

Basically, we deduced that the interaction between necroptosis-related miRNAs and certain genes may regulate these pathways triggering necroptosis, which eventually inference the progress of HNSCC.

## Supplementary Material

Supplementary Figure

Supplementary Table 1

Supplementary Table 2
