# Supplementary material for: Identification of a novel necroptosis-associated miRNA signature for predicting the prognosis in head and neck squamous cell carcinoma
Source: Open Med (Wars). 2022 Oct 25;17(1):1682–98. doi: 10.1515/med-2022-0575 (PMC9601379; doi:10.1515/med-2022-0575)
Supplement: Supplementary Figure [file med-2022-0575-SF.pdf]

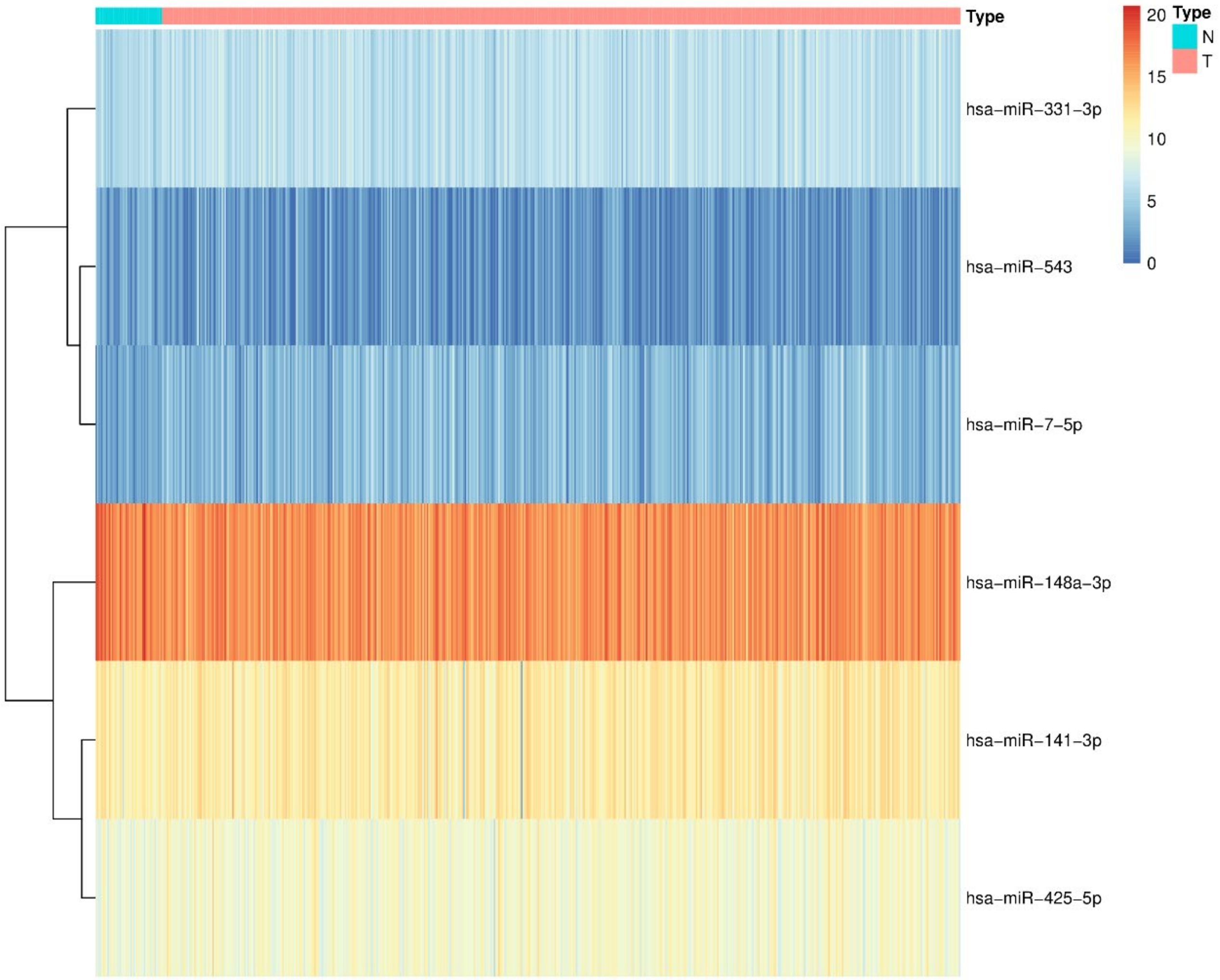

FigureS1 Expressions of the 6 differentially expressed necroptosis-related miRNAs. A Heatmap (blue: low expression level; red: high expression level) of the necroptosis-related miRNAs between the normal (N, green) and the tumor (T, orange) tissues.

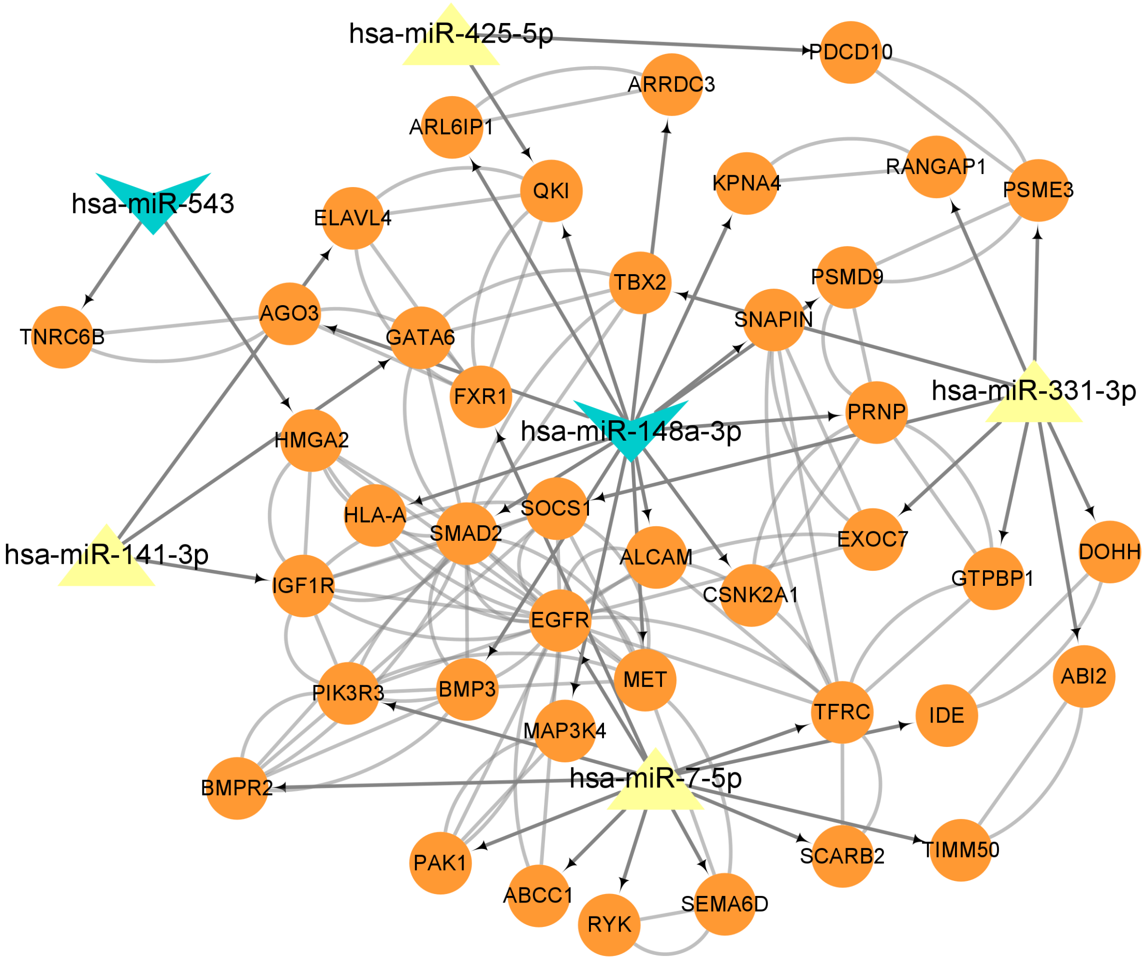

FigureS2 The regulatory network of the 6 prognostic miRNAs. Orange circle nodes: target genes; yellow triangle: up-regulated miRNAs; cyan V: down-regulated miRNAs; line without arrow: PPI relations; line with arrow: miRNA-gene regulating relations.

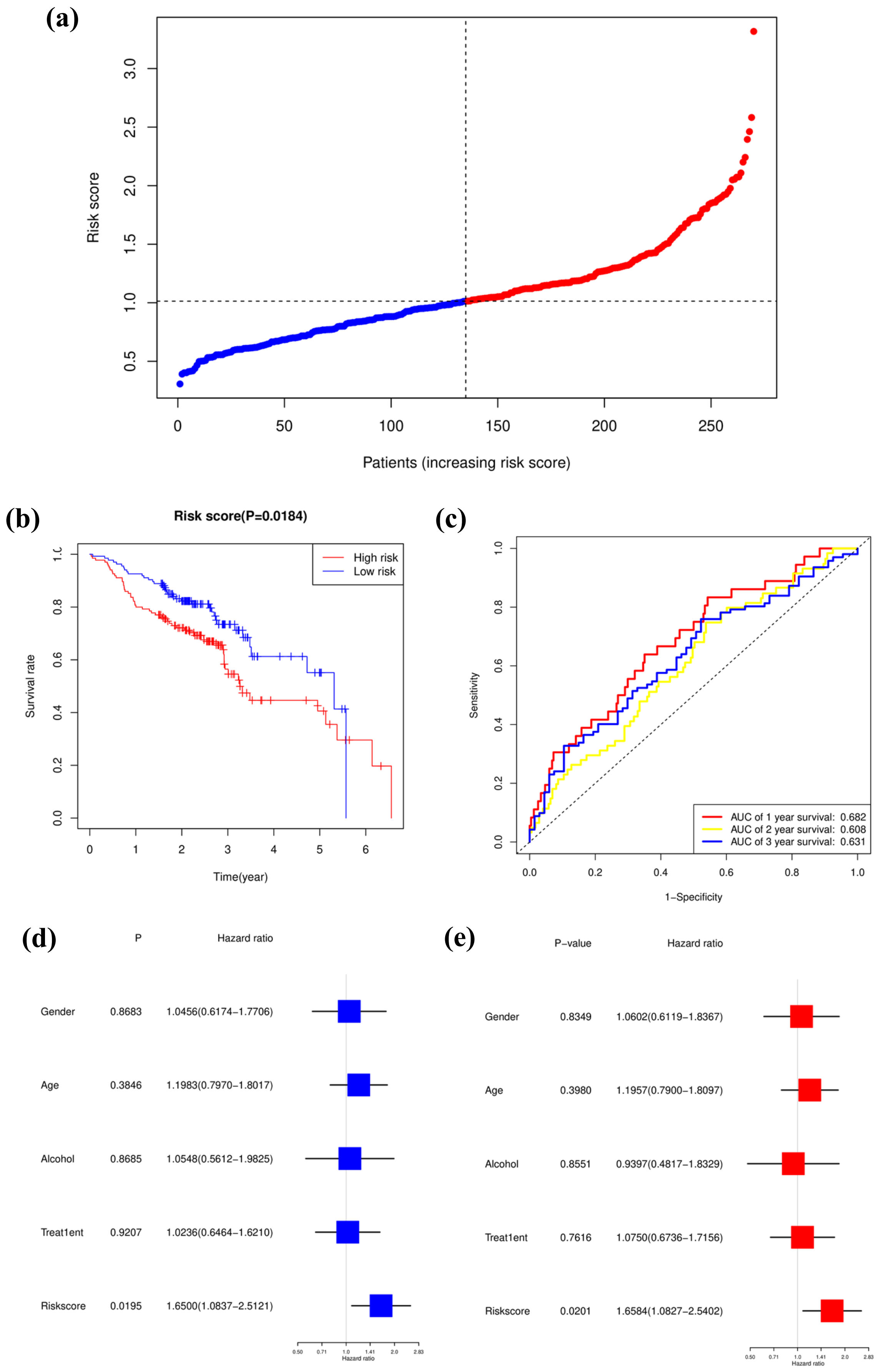

FigureS3 Validation of a prognostic model based on the key genes. (a) Distribution of patients based on the risk score. **(b)** Kaplan-Meier curves for the risks crore of patients in the high-risk and low-risk groups based on GEO database. **(c)** Time-dependent ROC curves for OCs. **(d)** Univariate cox regression analysis of OS for 5 risk-related factors. **(e)** Multivariate cox regression analysis of OS for 5 risk-related factors.
