# Supplementary material for: Identification of a novel necroptosis-associated miRNA signature for predicting the prognosis in head and neck squamous cell carcinoma
Source: Open Med (Wars). 2022 Oct 25;17(1):1682–98. doi: 10.1515/med-2022-0575 (PMC9601379; doi:10.1515/med-2022-0575)
Supplement: Supplementary Table 1 [file med-2022-0575-ST1.pdf]

Table S1: The clinical characteristics of patients in the TCGA and GEO dataset.

**Variable**	**Number of TCGA samples**	**Number of GEO samples**
Gender：		
Male/Female	386/142	223/47
Age at diagnosis：		
≤65/>65/NA	345/182/1	184/86
Stage：		
I/II/III/IV/NA	27/74/82/270/75	18/37/37/178/0
T：		
T0/T1/T2/T3/T4/NA	1/49/140/101/175/62	35/80/58/99/97/0
N：		
N0/N1/N2/N3/NA	180/68/172/8/100	94/32/132/12/0
M：		
M0/M1/NA	191/1/336	263/7/0
