# Supplementary material for: Identification of a novel necroptosis-associated miRNA signature for predicting the prognosis in head and neck squamous cell carcinoma
Source: Open Med (Wars). 2022 Oct 25;17(1):1682–98. doi: 10.1515/med-2022-0575 (PMC9601379; doi:10.1515/med-2022-0575)
Supplement: Supplementary Table 2 [file med-2022-0575-ST2.pdf]

**Table S2** Sixteen miRNAs known to be associated with necroptosis

| Necroptosis-associated miRNAs |
|---|
| miR-495 |
| miR-331-3p |
| miR-15a |
| miR-148a-3p |
| miR-7-5p |
| miR-141-3p |
| miR-425-5p |
| miR-200a-5p |
| miR-210 |
| miR-223-3p |
| miR-500a-3p |
| miR-181-5p |
| miR-16-5p |
| miR-371-5p |
| miR-373 |
| miR-543 |
